# Supplementary figures and images for: Genome editing to model and reverse a prevalent mutation associated with myeloproliferative neoplasms
Source: PLoS One. 2021 Mar 4;16(3):e0247858. doi: 10.1371/journal.pone.0247858 (PMC7932127; doi:10.1371/journal.pone.0247858)

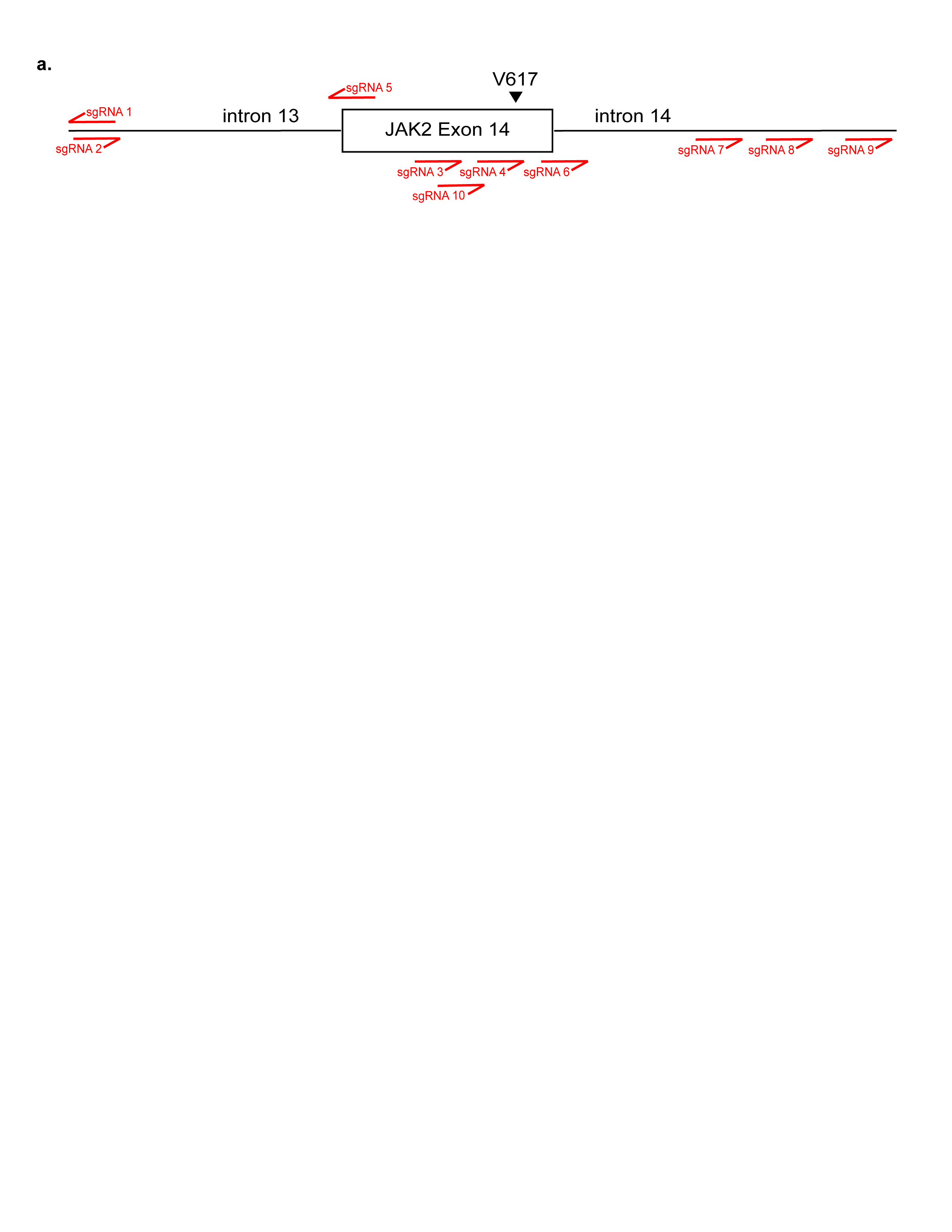

Supplement: S1 Fig — (a) Schematic indicating positions of all sgRNAs designed and tested. (TIF) [file pone.0247858.s001.tif]

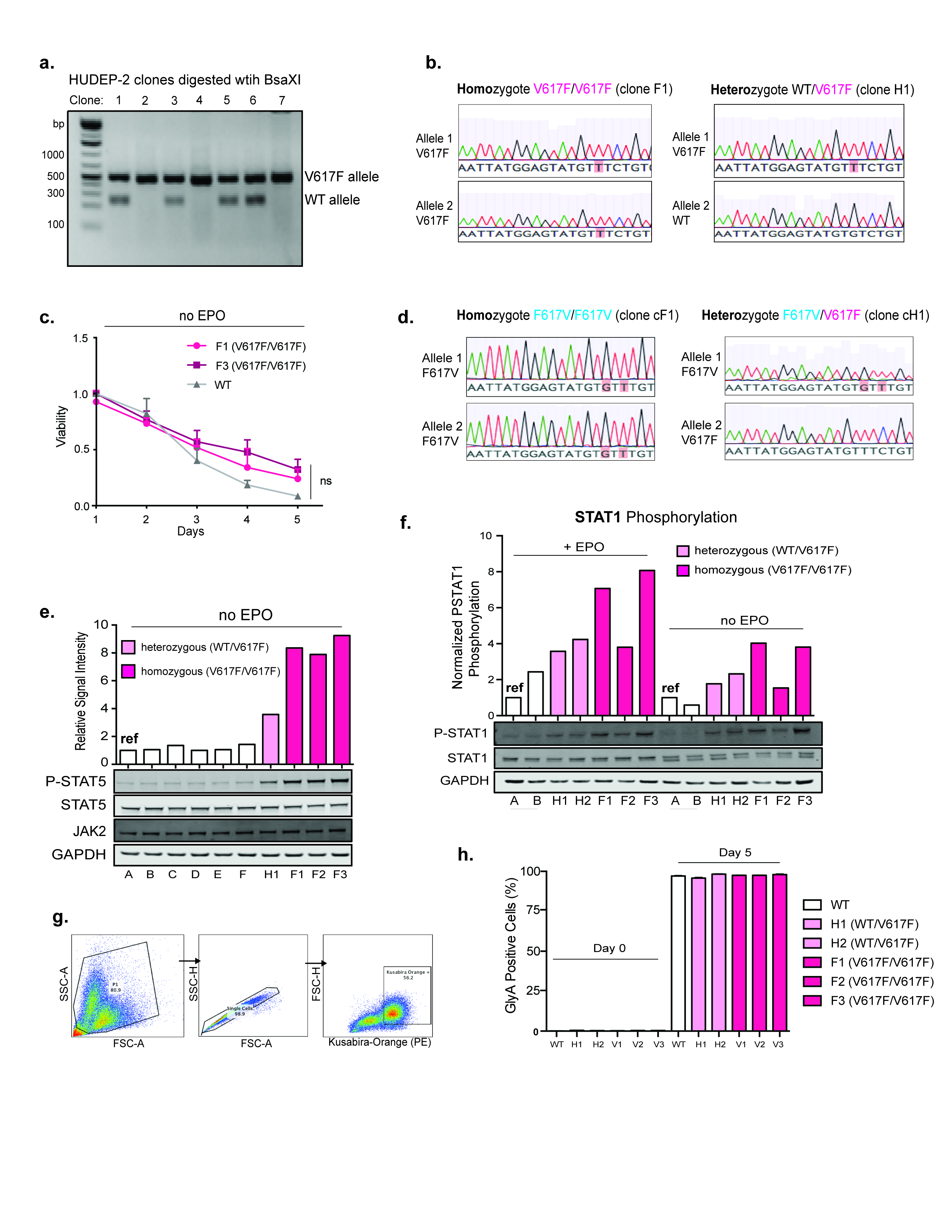

Supplement: S2 Fig — (a) DNA gel showing screening of V617F clones by PCR and BsaXI restriction digest. Lower band corresponds to WT allele and higher undigested fragment corresponds to V617F allele. (b) Sanger sequencing traces of V617F homozygous (F1) and heterozygous (H1) clones. (c) Viability curve depicting cell death in the absence of EPO. F1 and F3 V617F homozygote clones exhibited mildly higher cell viability than WT cells. Data is from n = 5 independent biological replicates. Mean of all experiments ± SD shown. (d) Sanger sequencing traces of F617V homozygous (cF1) and heterozygous (cH2) clones. (e) Immunoblot and signal intensity quantification show elevated phosphorylated STAT5 (P-STAT5) and uniform JAK2 expression in JAK2 V617F HUDEP-2 clones without erythropoietin (EPO). Signals were normalized to STAT5 and GAPDH. White bars, JAK2 WT clones; light pink bar, JAK2 V617F heterozygous clone; magenta bars, JAK2 V617F homozygous clones. (f) Immunoblot and signal intensity quantification show elevated phosphorylated STAT1 (P-STAT1) expression in JAK2 V617F HUDEP-2 clones both with and without erythropoietin (EPO). Signals were normalized to STAT1 and GAPDH. White bars, JAK2 WT clones; light pink bars, JAK2 V617F heterozygous clones; magenta bars, JAK2 V617F homozygous clones. (g) Representative FACS plots for gating live HUDEP-2s expressing Kusabira Orange, a marker gene indicative of viable HUDEP-2s. (h) Levels of Glycophorin A (GlyA), an erythroid-specific cell surface marker, in undifferentiated or fully differentiated HUDEP-2s at day 0 and day 5, respectively. (TIF) [file pone.0247858.s002.tif]

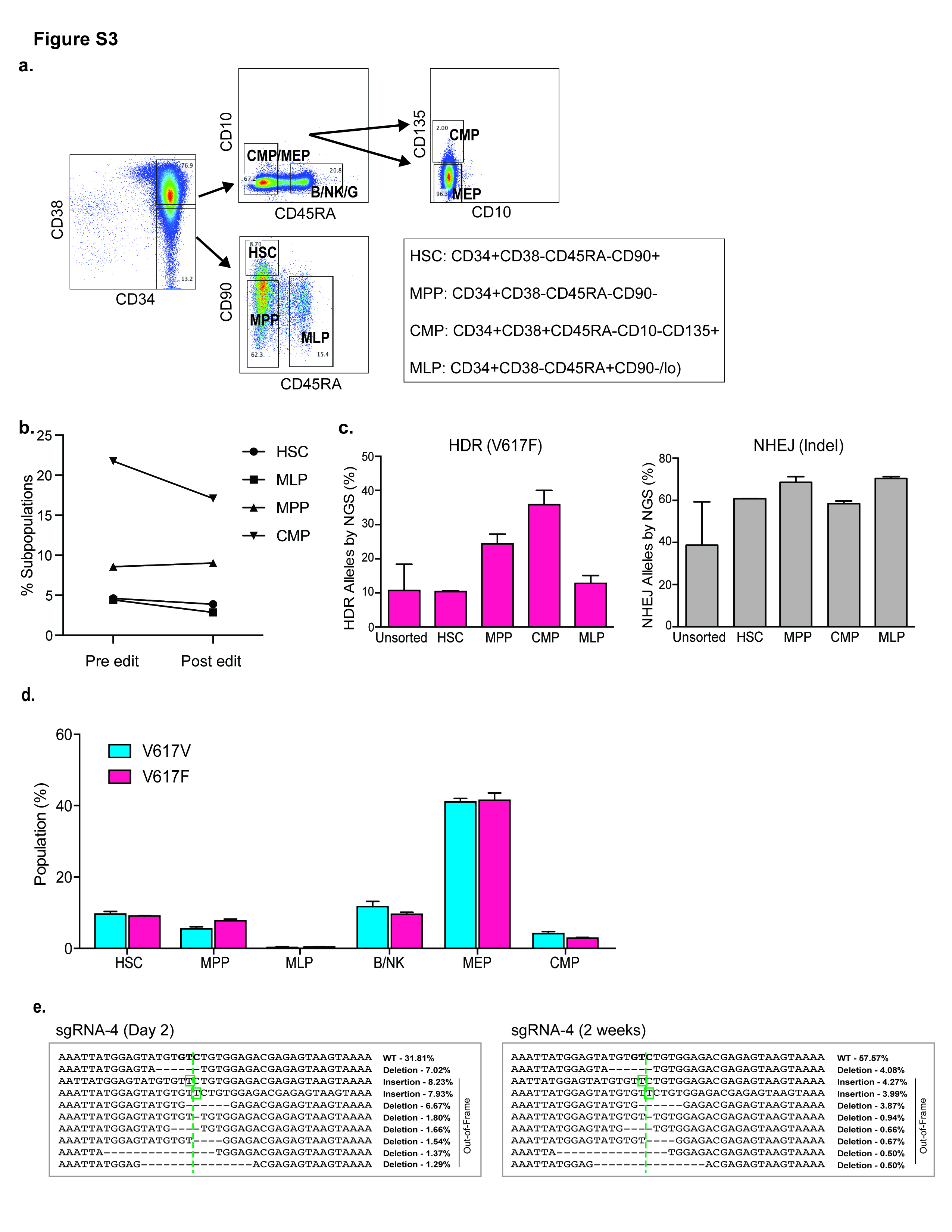

Supplement: S3 Fig — (a) Gating schematic for subsets of CD34+ HSPCs including HSCs (CD34+ CD38- CD45RA- CD90+), multipotent progenitors (MPPs) (CD34+ CD38- CD45RA- CD90-), multipotent lymphoid progenitors (MLPs) (CD34+ CD38- CD45RA+ CD90-/lo), common myeloid progenitors (CMPs) (CD34+ CD38+ CD45RA- CD10- CD135+), megakaryocyte-erythroid progenitors (MEPs) (CD34+ CD38+ CD45RA- CD10- CD135-), and B/NK cells (CD34+ CD38+ CD45RA+ CD10-). (b) Composition of HSPC subsets (HSC, MLP, MPP, and CMP) in culture at the time of edit and 3 days post-edit as determined by flow cytometry using gating strategy described in (a). (c) HDR-mediated outcomes in HSPC subsets were assessed by amplicon-NGS 3 days after electroporation. Data from n = 3 independent biological replicates. Mean±SD shown. (d) NHEJ-mediated outcomes of cells in (c) were assessed by amplicon-NGS 3 days after electroporation. Data from n = 3 biological replicates. Mean±SD shown. (e) Fraction of CD34+ HSPC subpopulations in V617F or V617V edited CD34+ bulk cells after 4 days of edit. Data from n = 2 independent biological replicates. Mean±SD shown. (TIF) [file pone.0247858.s003.tif]

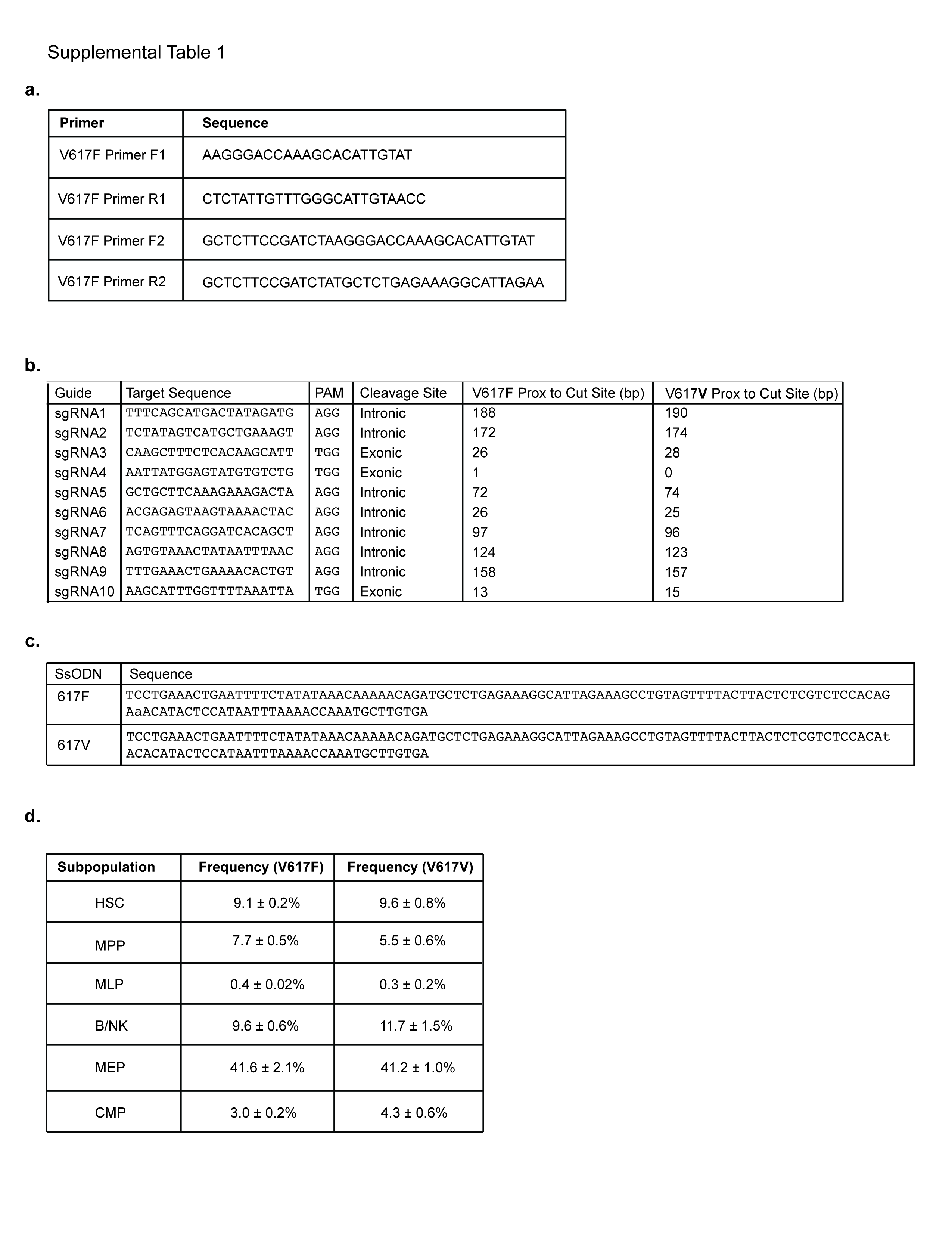

Supplement: S1 Table — (a) Protospacer sequences and proximities to target site of guides shown in. (b) Sequences of locus-specific primer sets used for T7E1 assay, clonal screening, and amplicon-NGS. (c) Sequences of ssODNs used to generate 617V and 617F mutations. (TIF) [file pone.0247858.s004.tif]
