# Supplementary material for: Genome editing to model and reverse a prevalent mutation associated with myeloproliferative neoplasms
Source: PLoS One. 2021 Mar 4;16(3):e0247858. doi: 10.1371/journal.pone.0247858 (PMC7932127; doi:10.1371/journal.pone.0247858)

Acquisition Information

#	Image ID	Acquire Time	Channels	Resolution	Intensities	Image Name	Comment
1	0003282_01	Aug 10, 2018 2:19:53 PM	700 800	169um	Auto Auto	Blot 1: PSTAT5, GAPDH and Bglobin	HUDEPs (100% and 0% Epo)

Image Display Values

Channel	Color	Minimum	Maximum	K
800	Gray Scale (Black on White)	0.0482	3.59	0

Figure 2c PSTAT5 and GAPDH and Bglobin

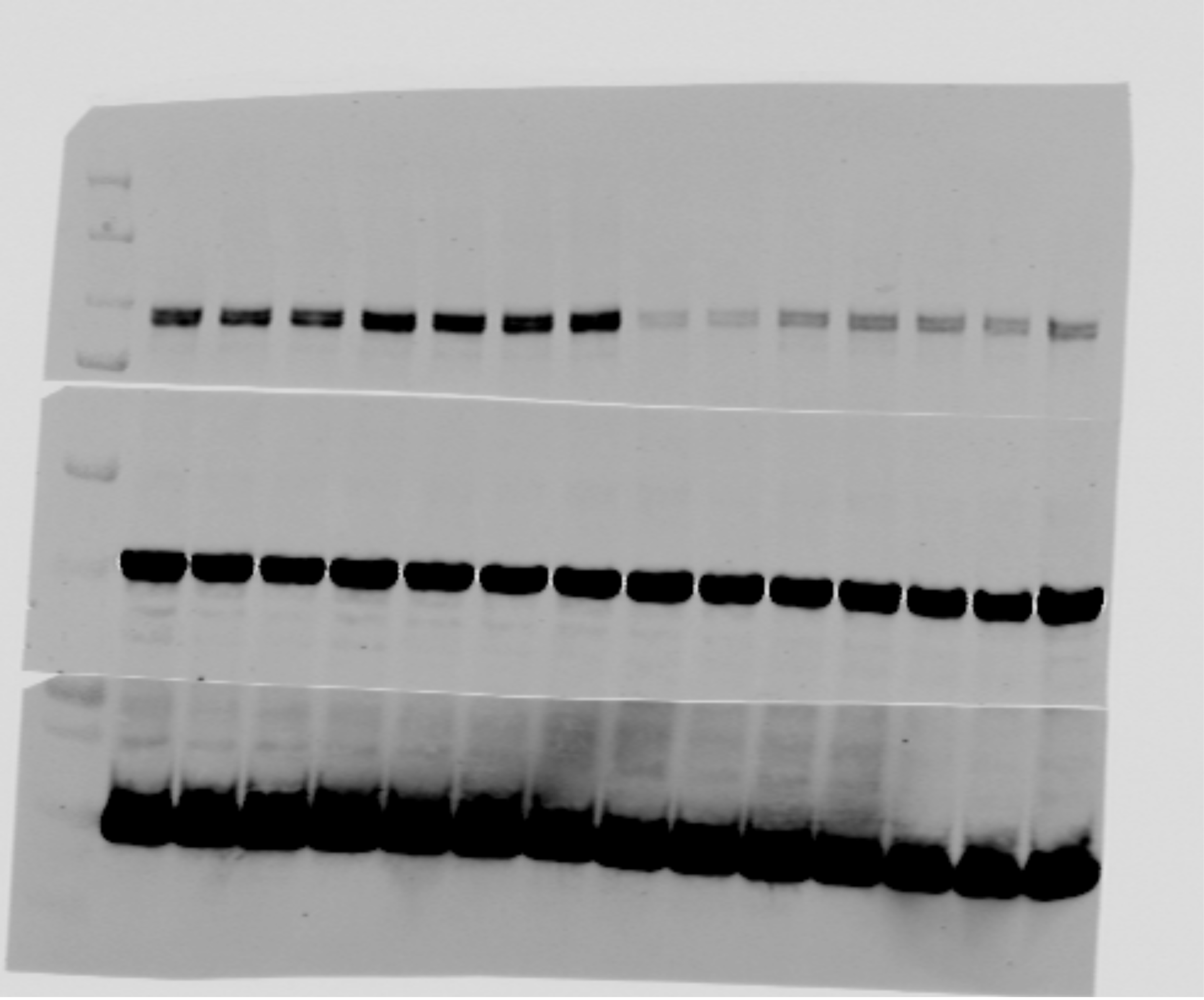

Image ID: 0003284_01
Acquire Time: Aug 10, 2018 2:34:48 PM

Acquisition Information

#	Image ID	Acquire Time	Channels	Resolution	Intensities	Image Name	Comment
1	0003284_01	Aug 10, 2018 2:34:48 PM	700 800	169um	Auto Auto	Blot 3: STAT5, GAPDH, and Bglobin	HUDEPs (100% and 0% Epo)

Image Display Values

Channel	Color	Minimum	Maximum	K
800	Gray Scale (Black on White)	0.688	7.42	0

Figure 2c STAT5 and GAPDH and Bglobin

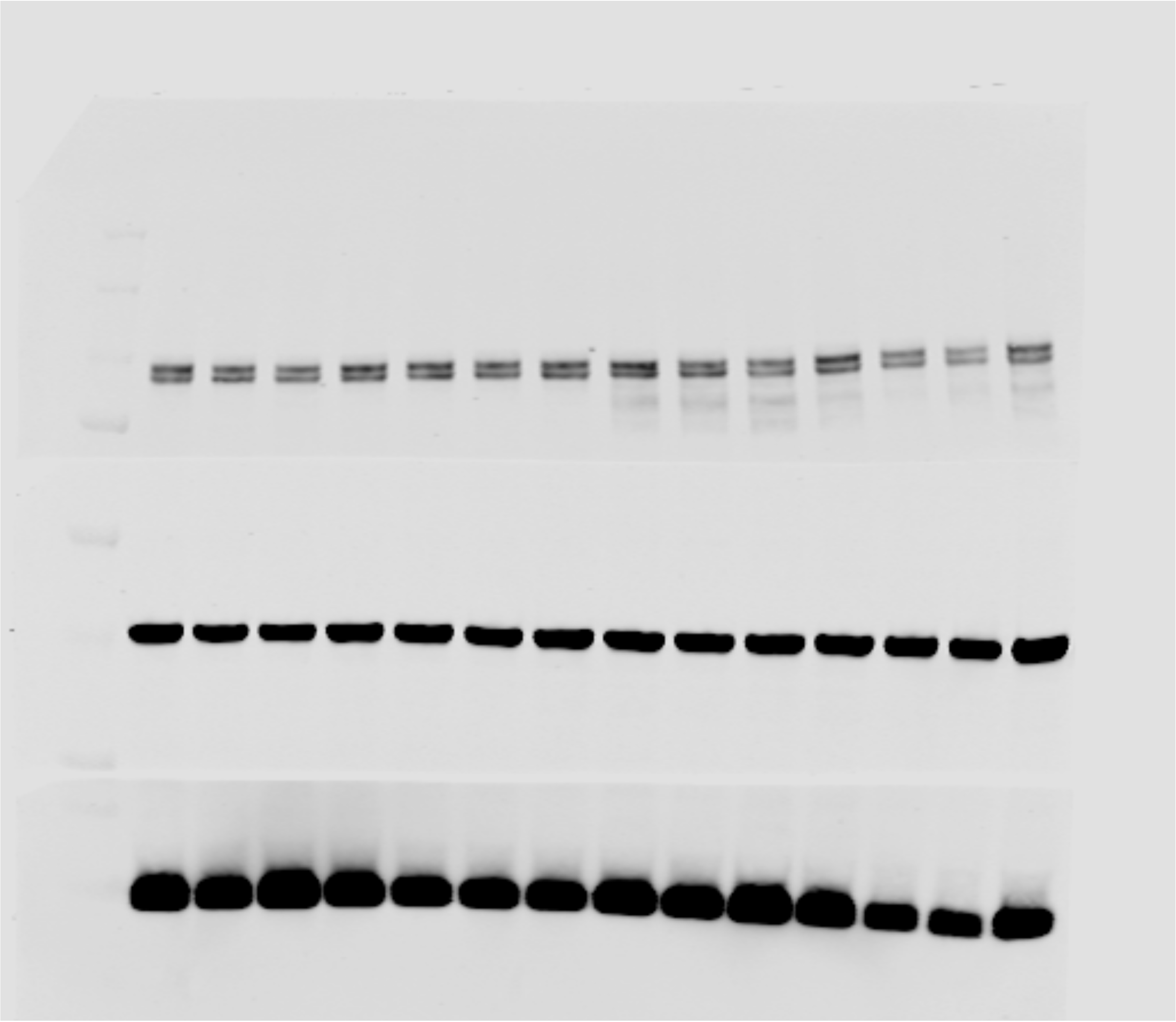

Acquisition Information

#	Image ID	Acquire Time	Channels	Resolution	Intensities	Image Name	Comment
1	0003689_01	Sep 28, 2019 1:59:35 PM	700 800	169um	Auto Auto	Blot1: PSTAT5 and GAPDH (no epo) good blot	

Image Display Values

Channel	Color	Minimum	Maximum	K
800	Gray Scale (Black on White)	0.222	2.98	0

Figure 2d PSTAT5 and GAPDH and Bglobin

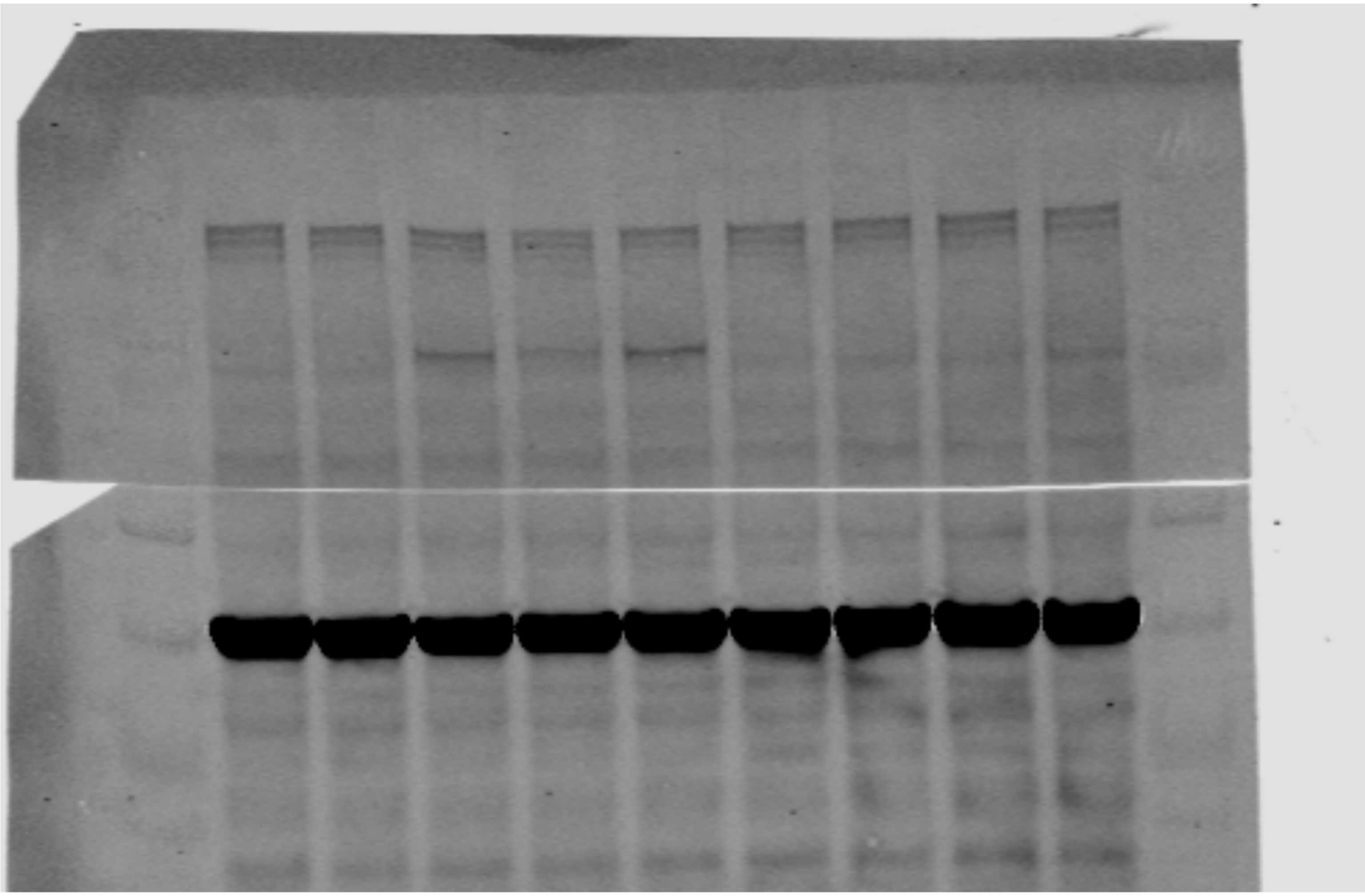

Acquisition Information

#	Image ID	Acquire Time	Channels	Resolution	Intensities	Image Name	Comment	Image Modifications
1	0003690_01	Sep 28, 2019 2:05:10 PM	700 800	169um	Auto Auto	Blot 2: STAT5 anf GAPDH (no epo)		Flip

Image Display Values

Channel	Color	Minimum	Maximum	K
800	Gray Scale (Black on White)	0.0183	1.87	0

Figure 2d STAT5 and GAPDH

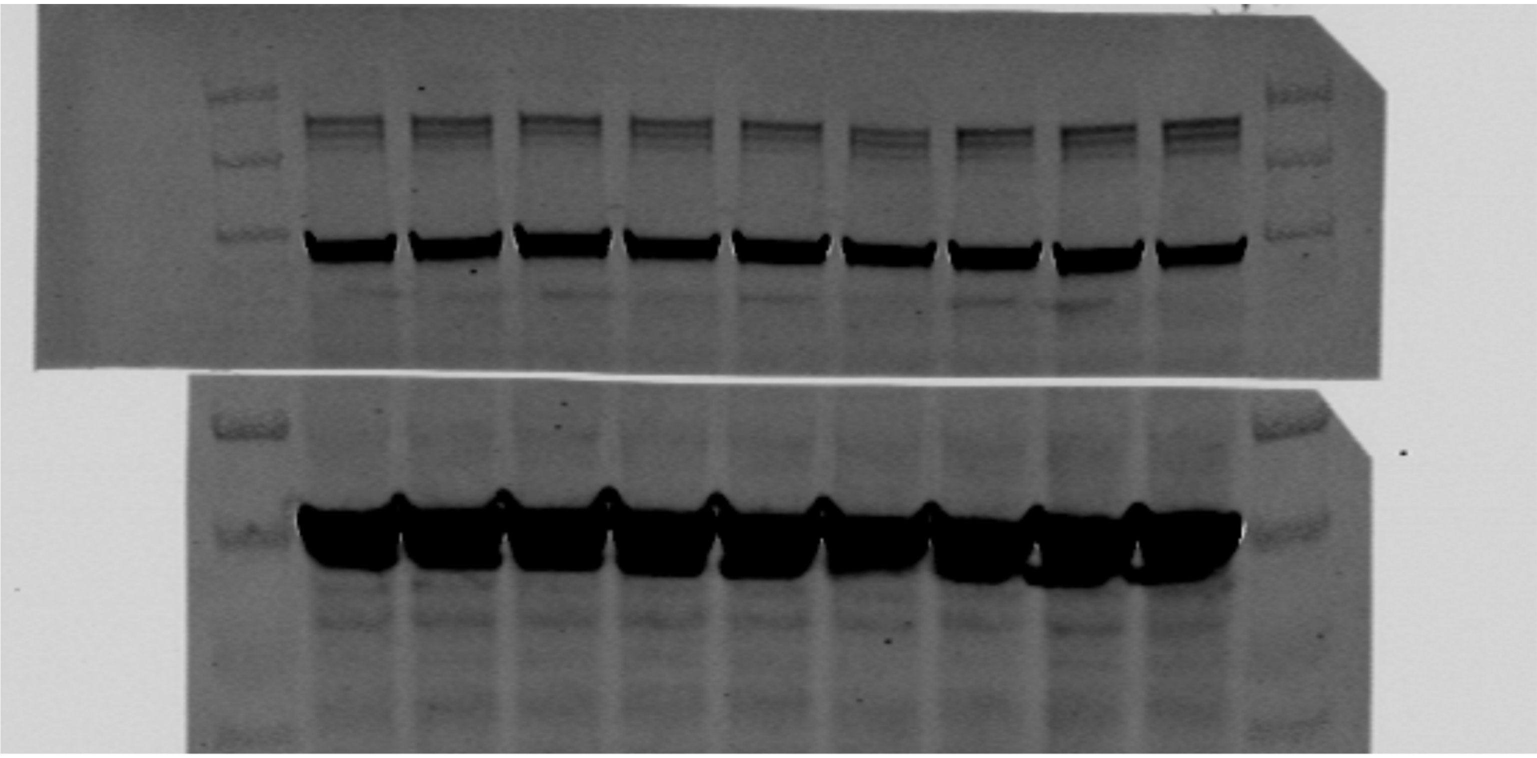

LI-COR®

Image ID: 0002310_01
Acquire Time: Mar 18, 2018 6:26:17 PM

Acquisition Information

#	Image ID	Acquire Time	Channels	Resolution	Intensities	Image Name
1	0002310_01	Mar 18, 2018 6:26:17 PM	700 800	169um	Auto Auto	Blot 4: PSTAT5, GAPDH and Bglobin flipped

Figure 4c PSTAT5 and GAPDH and Bglobin. *This blot has been cut and reversed as shown below*

Image Display Values

Channel	Color	Minimum	Maximum	K
800	Gray Scale (Black on White)	0.0391	2.23	0

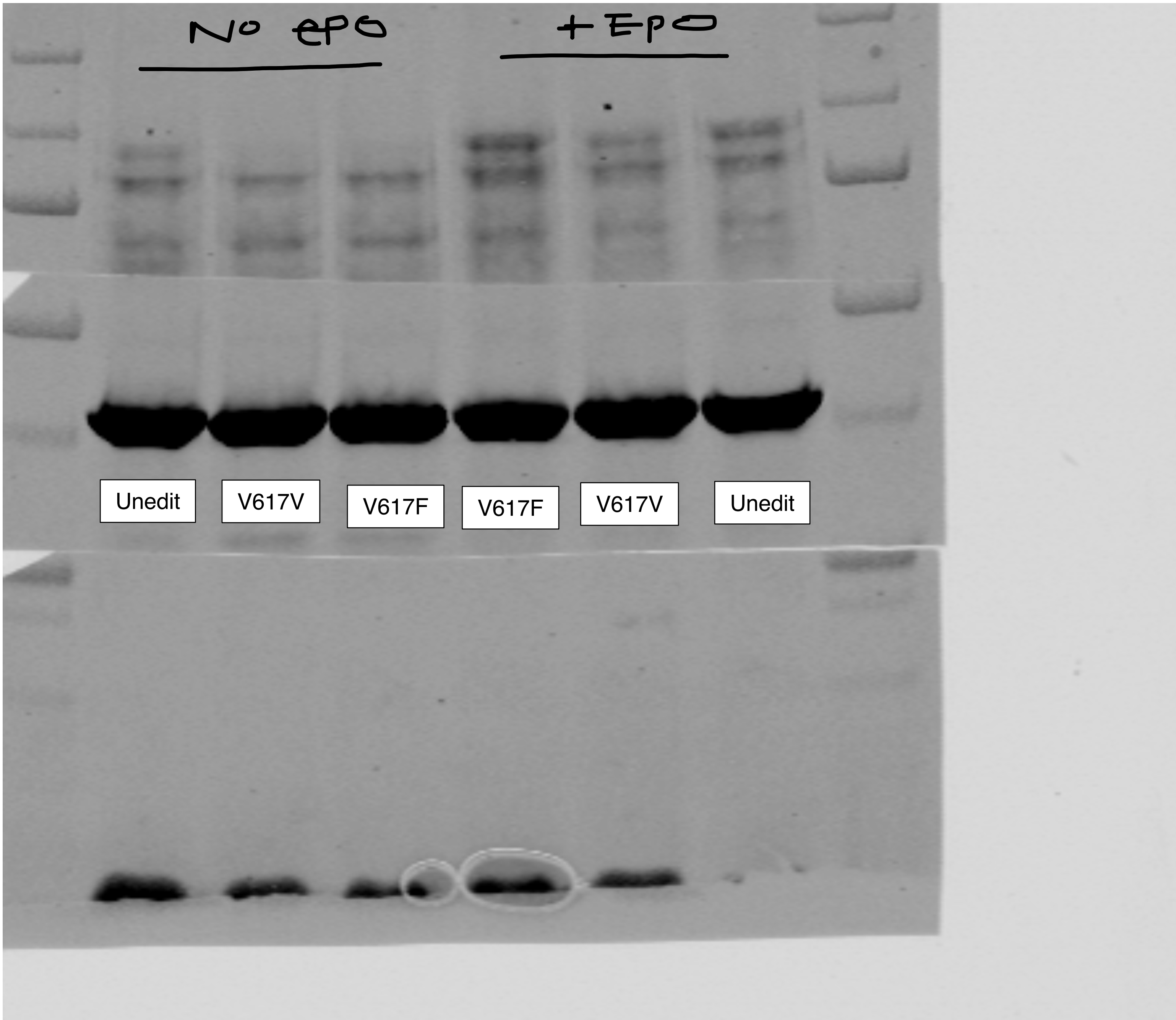

LI-COR®

Image ID: 0002307_02
Acquire Time: Mar 18, 2018 6:06:59 PM

Acquisition Information

#	Image ID	Acquire Time	Channels	Resolution	Intensities	Image Name	Comment
1	0002307_02	Mar 18, 2018 6:06:59 PM	700 800	169um	Auto Auto	Blot 2: STAT5, GAPDH, Bglobin	new HSC colonies (100% and Epo)

Image Display Values

Channel	Color	Minimum	Maximum	K
700	Gray Scale (Black on White)	3.15	326	0
800	Gray Scale (Black on White)	0.821	1.38	0

Figure 4c STAT5 and GAPDH and Bglobin.

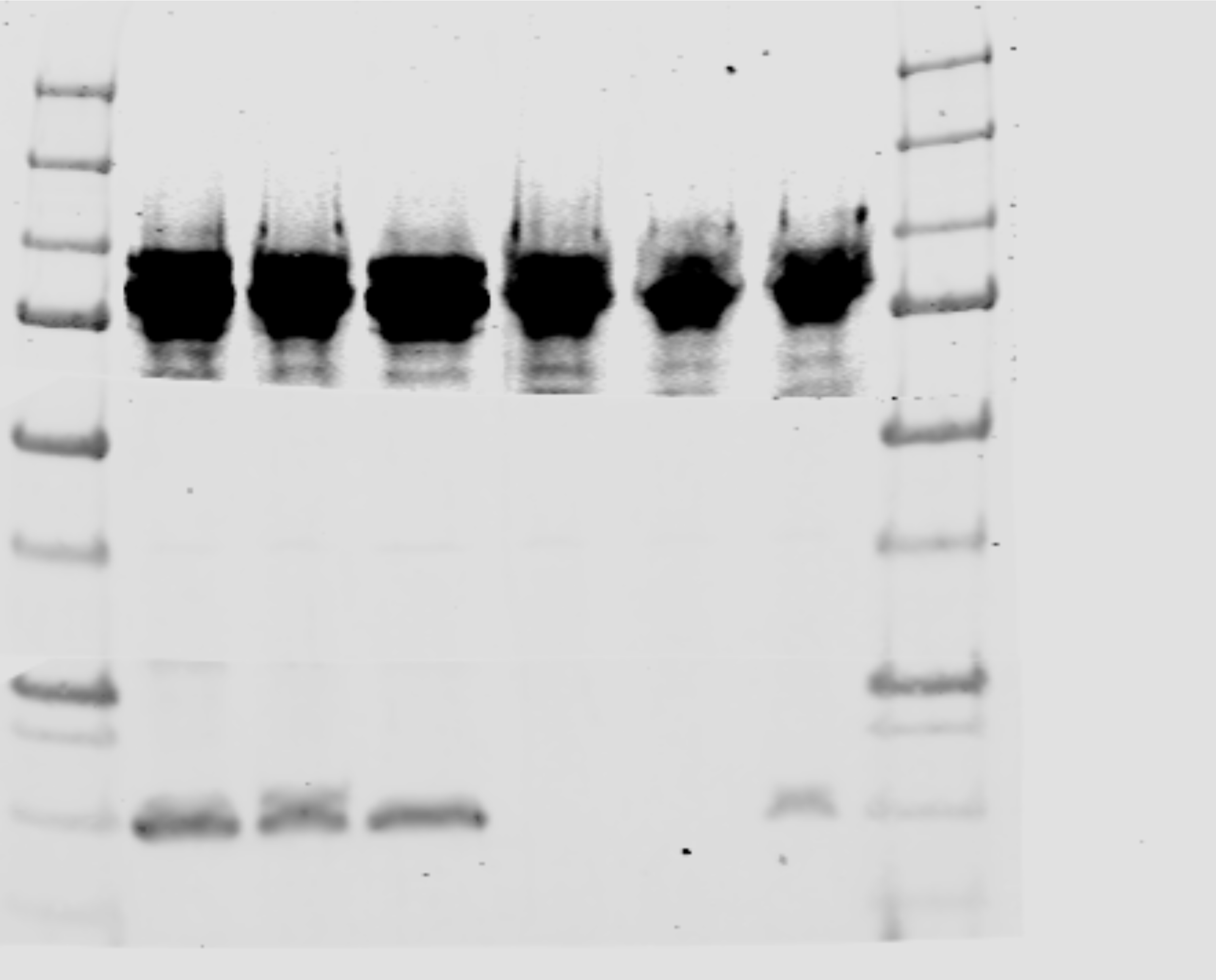

Acquisition Information

#	Image ID	Acquire Time	Channels	Resolution	Intensities	Image Name	Comment
1	0001536_01	Dec 5, 2017 12:50:37 AM	700 800	169um	Auto Auto	HUDEP 0% Epo; JAK2 (reblot on PSTAT5 blot) membrane 2	

Image Display Values

Channel	Color	Minimum	Maximum	K
700	Gray Scale (Black on White)	0.350	5.06	0
800	Gray Scale (Black on White)	0.786	1.02	0

Figure S2e JAK2

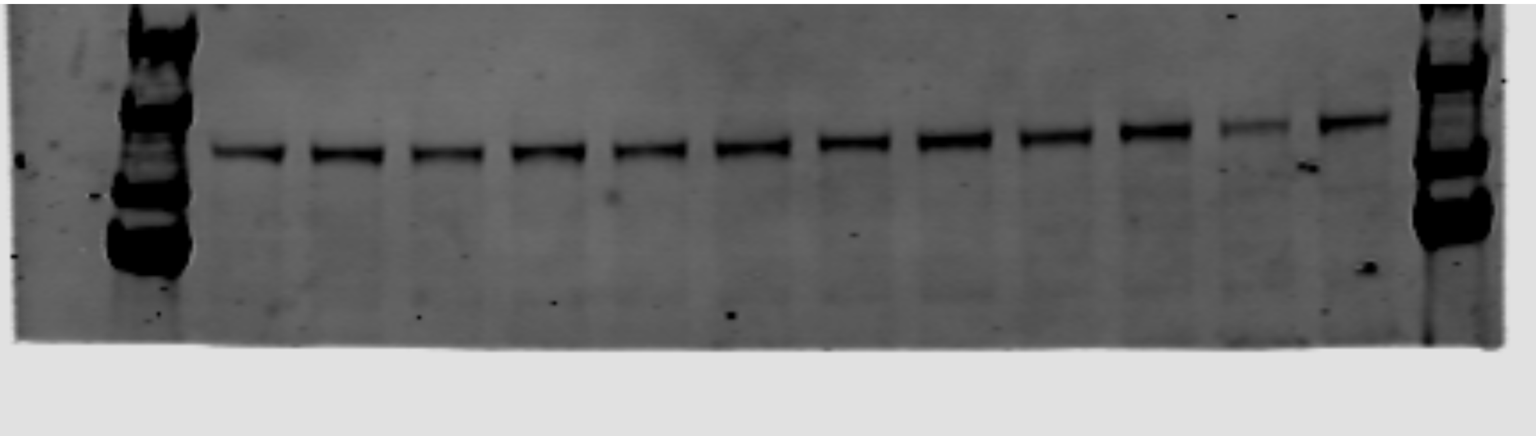

Acquisition Information

#	Image ID	Acquire Time	Channels	Resolution	Intensities	Image Name	Comment	Image Modifications
1	0001512_01	Dec 1, 2017 10:35:28 PM	700 800	169um	Auto Auto	HUDEP 0% Epo; PSTAT5 and GAPDH		

Image Display Values

Channel	Color	Minimum	Maximum	K
700	Gray Scale (Black on White)	3.54	102	0
800	Gray Scale (Black on White)	0.0916	3.80	0

Figure S2e PSTAT5 and GAPDH

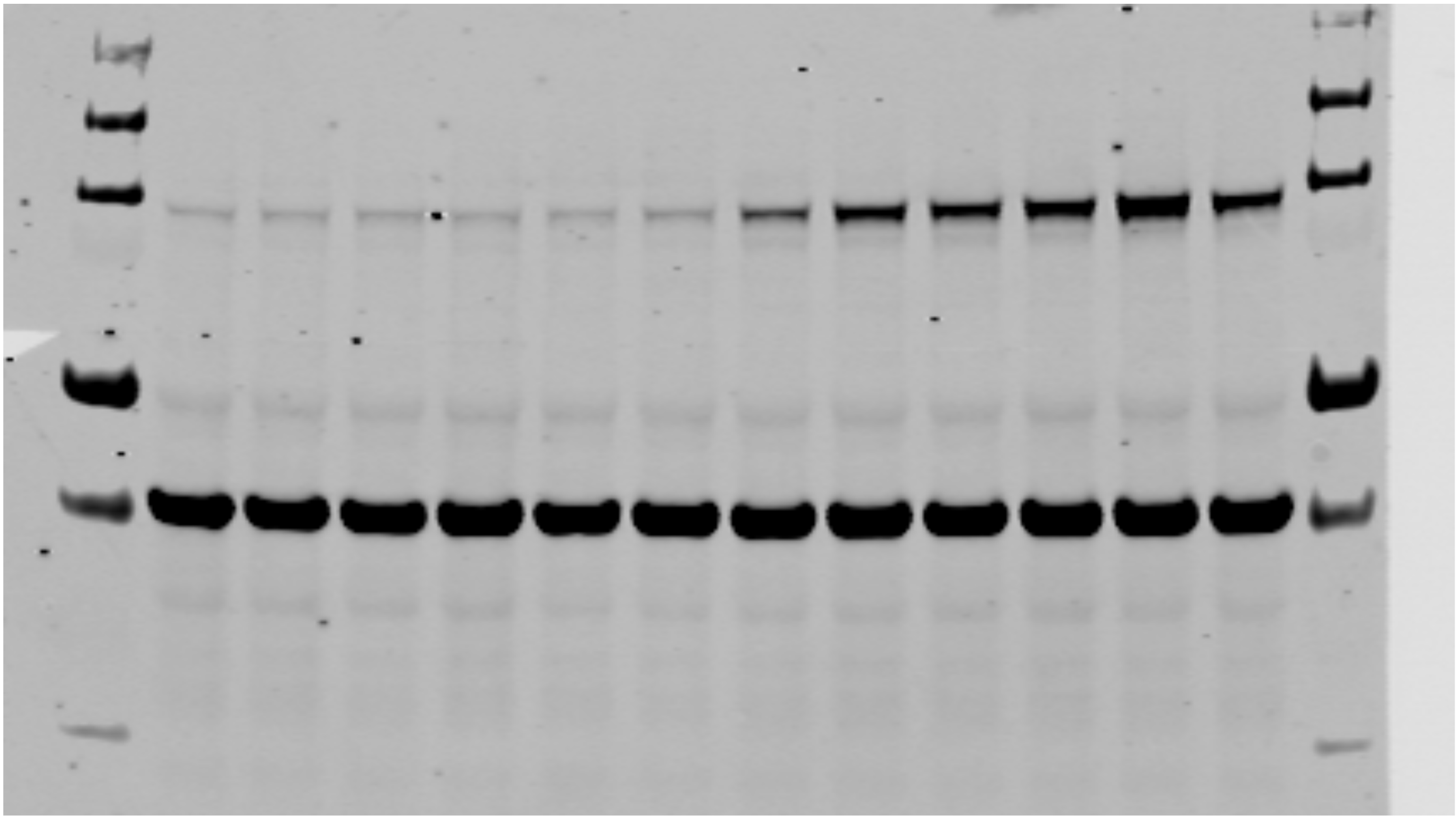

Acquisition Information

#	Image ID	Acquire Time	Channels	Resolution	Intensities	Image Name	Comment	Image Modifications
1	0001511_01	Dec 1, 2017 10:30:42 PM	700 800	169um	Auto Auto	HUDEP 0% Epo; STAT5 and GAPDH		

Image Display Values

Channel	Color	Minimum	Maximum	K
700	Gray Scale (Black on White)	0.488	19.0	0
800	Gray Scale (Black on White)	0.759	51.6	0

Figure S2e STAT5 and GAPDH

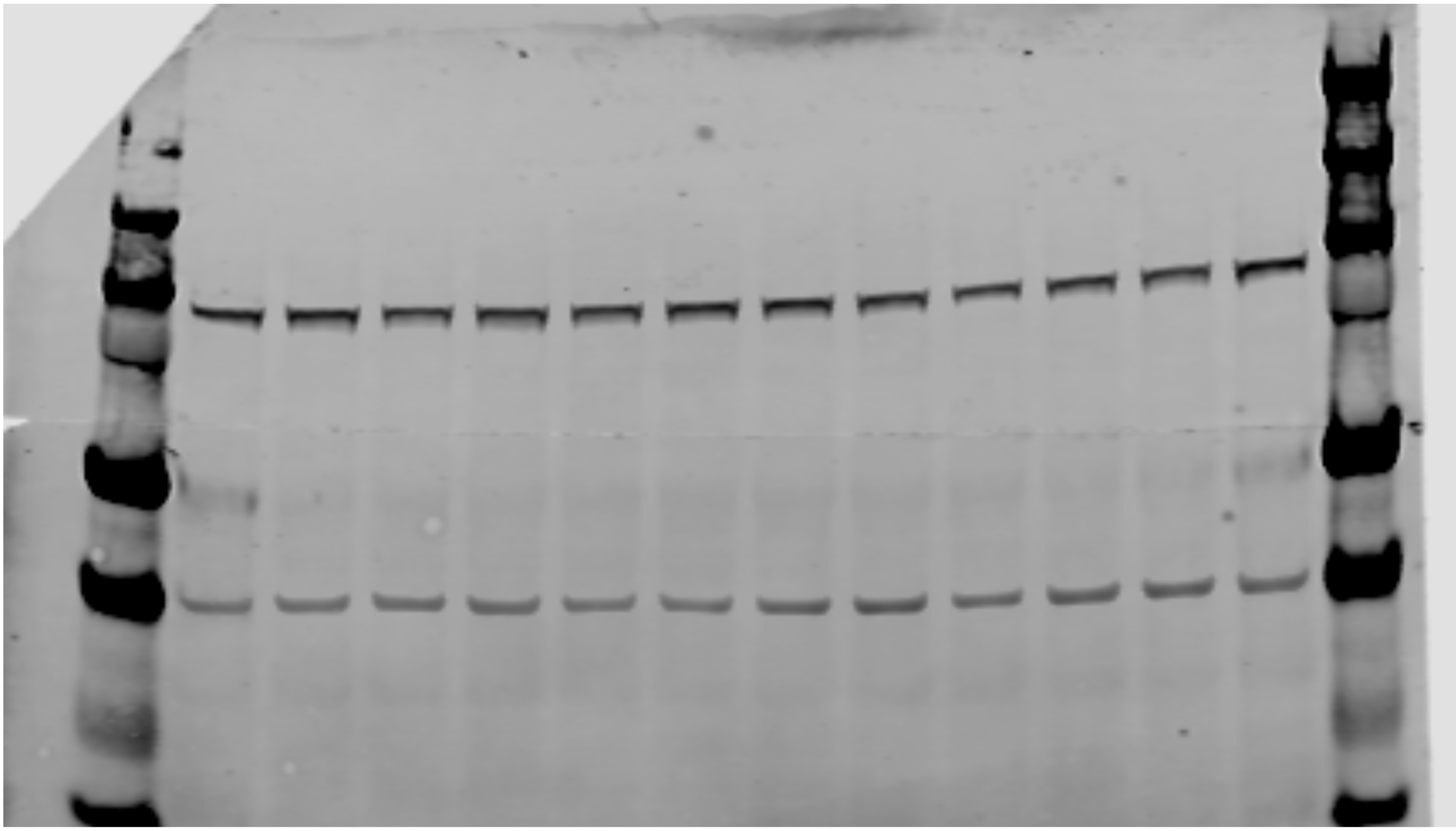

Image ID: 0003283_01
Acquire Time: Aug 10, 2018 2:27:30 PM

Acquisition Information

#	Image ID	Acquire Time	Channels	Resolution	Intensities	Image Name	Comment
1	0003283_01	Aug 10, 2018 2:27:30 PM	700 800	169um	Auto Auto	Blot 2: PSTAT1, GAPDH and Bglobin	HUDEPs (100% and 0% Epo)

Image Display Values

Channel	Color	Minimum	Maximum	K
700	Gray Scale (Black on White)	0.103	191	0
800	Gray Scale (Black on White)	0.169	1.62	0

Figure S2f PSTAT1 and GAPDH and Bglobin

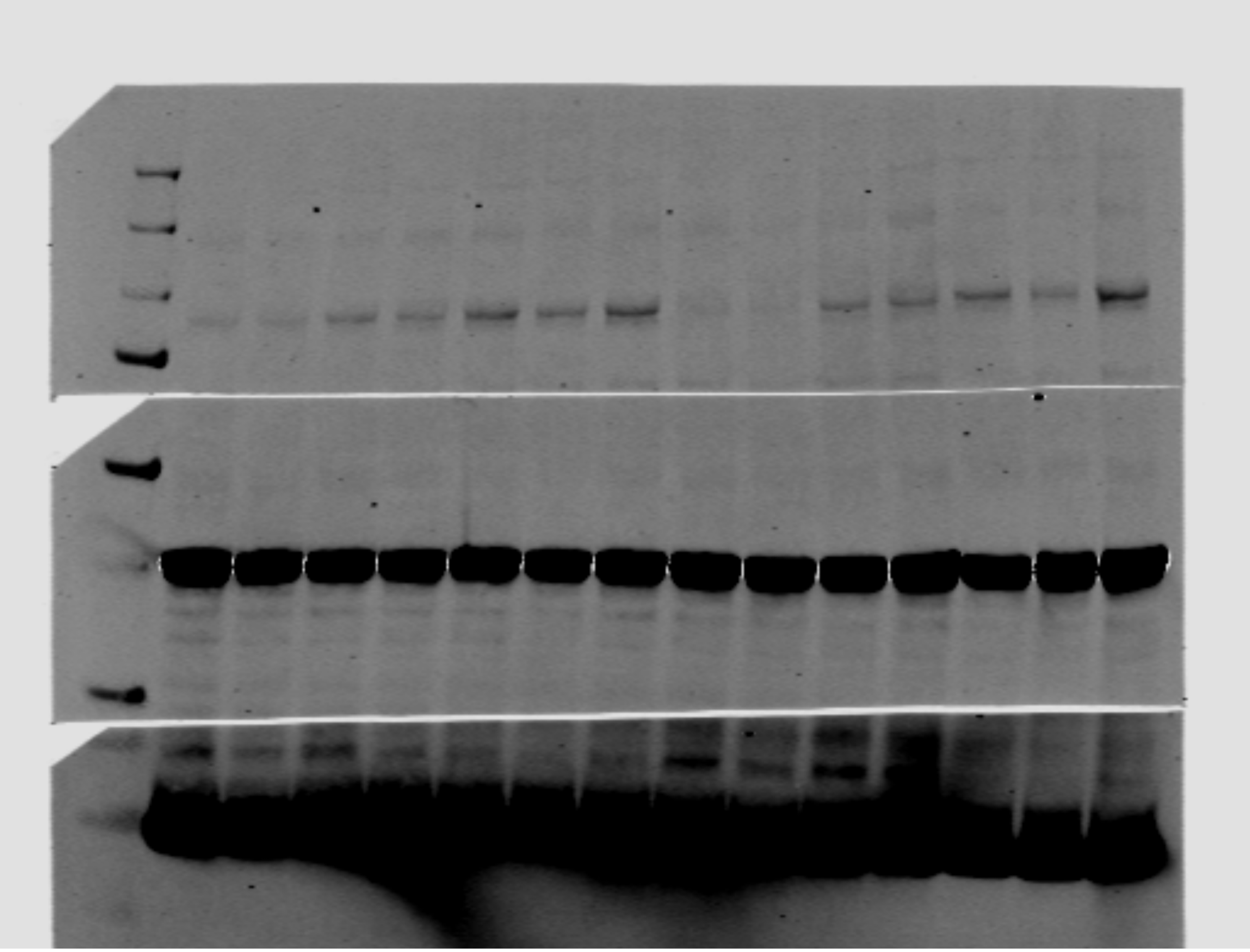

Image ID: 0003285_01
Acquire Time: Aug 10, 2018 2:42:29 PM

Acquisition Information

#	Image ID	Acquire Time	Channels	Resolution	Intensities	Image Name
1	0003285_01	Aug 10, 2018 2:42:29 PM	700 800	169um	Auto Auto	Blot 4: STAT1, GAPDH and Bglobin

Image Display Values

Channel	Color	Minimum	Maximum	K
800	Gray Scale (Black on White)	0.304	2.10	0

Figure S2f STAT1 and GAPDH and Bglobin

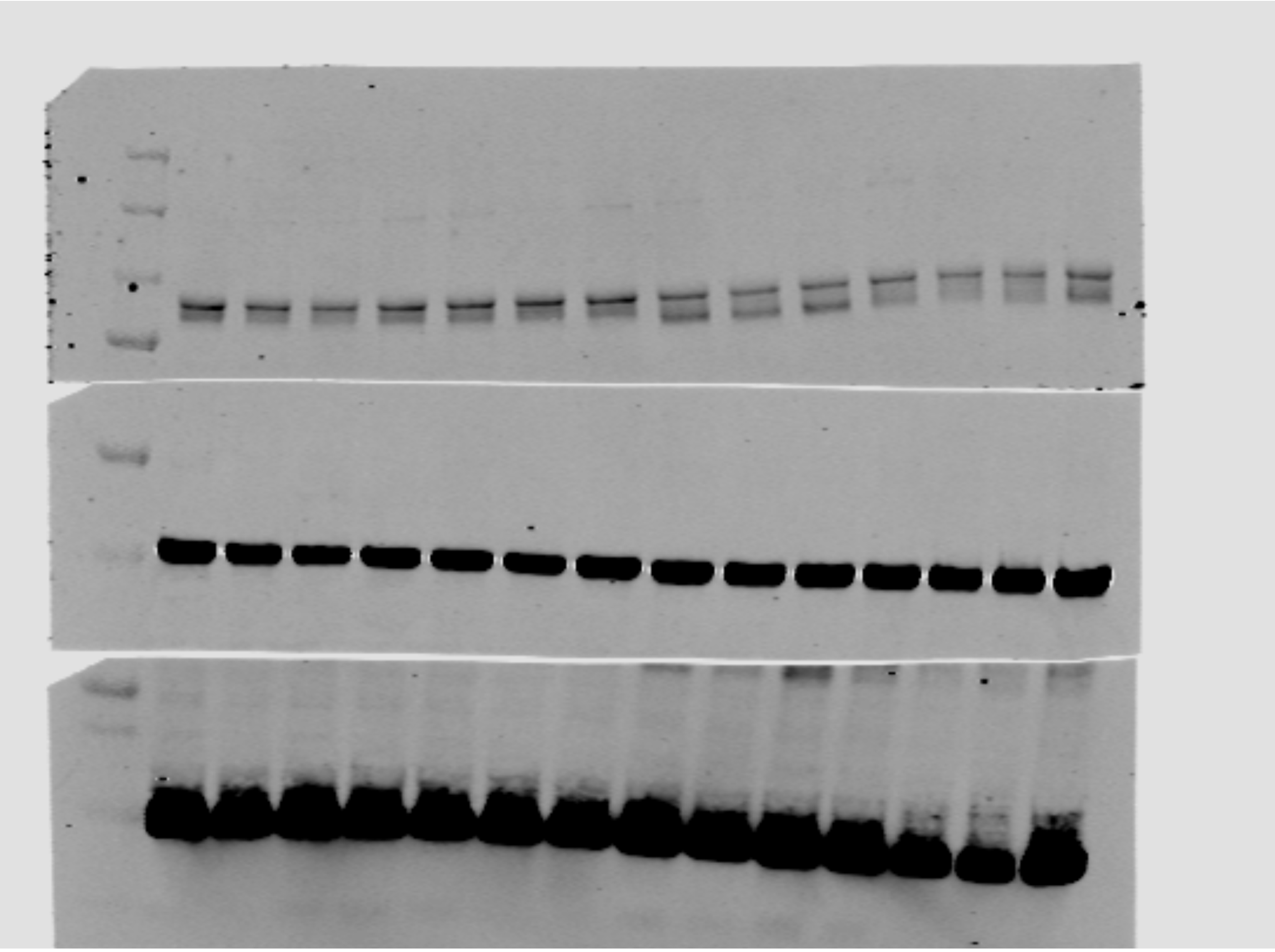

Supplement: S1 Raw images — (PDF) [file pone.0247858.s005.pdf]
