# Supplementary material for: Genome editing to model and reverse a prevalent mutation associated with myeloproliferative neoplasms
Source: PLoS One. 2021 Mar 4;16(3):e0247858. doi: 10.1371/journal.pone.0247858 (PMC7932127; doi:10.1371/journal.pone.0247858)
Supplement: Attachment — Submitted filename: eLife JAK2 V617F Referee Rebuttal.docx [file pone.0247858.s006.docx]

Reviewer #1 (General assessment and major comments (Required)):

In this manuscript Baik et al. generate a genome editing model of JAK2-V617F mutations using CRISPR-Cas9 and ssDNA oligonucleotides. They demonstrate successful editing in cell lines as well as primary human CD34+ HSPCs. In HUDEP-2 cell lines, they argue that V617F results in very little measurable cell autonomous changes, but confers a growth advantage in the context of co-culture with WT cells. In primary cells, they find that V617F edited CD34+ HSPCs are capable of differentiating into mature, beta-globin expressing erythroid cells in the absence of EPO. Finally, they establish reagents and demonstrate correction of the V617F mutation in primary CD34+ cells. Overall, the use of genome editing in primary human HSPCs is of interest, however, there are several issues with this manuscript:

1. The manuscript describes F617V reversion experiments that rescue wild type protein expression and discuss this as a potential therapeutic strategy. However, as the authors note in the discussion, therapeutic genome editing of mutations associated with oncogenic transformation is unlikely to succeed due to the fitness advantage of even small numbers of uncorrected cells. Instead of focusing on F617V reversion as a therapy, perhaps the reagents can be framed as rigorous controls for technically challenging experiments.

The referee is entirely correct that our primary intention with the F617V reversion reagents was to act as a “clean” control. We apologize if this was not clear. We feel that these reversion editing reagents are important in two ways. First, we edit wild type cells and show that the phenotypes observed are not a result of editing itself (e.g. due to p53 activation). Second, we revert the V617F mutation (itself made using genome editing), which is akin to an endogenous rescue experiment. We included discussion (in lines 432-445) of the difficulties involved in therapeutic genome editing of oncogenic mutations to avoid over-promising the potential clinical utility of these reagents. We have been contacted by the MPN Foundation and MPN patients about the possibility of therapeutic genome editing of JAK2 V617F. Hence, we feel it important to acknowledge the theoretical utility of such reagents together with the concerns that may present a practical barrier.

2. As the authors note, it is important to use multiple single-cell derived genome edited cell lines in order to support strong conclusions. However, many of the experiments in Figure 2e-h seem to be based on a single WT and only two V617F lines. Due to the small numbers of lines being compared, it is not clear that the experiments are statistically powered to demonstrate non-inferiority, for example that V617F does not results in a cell autonomous increase in proliferation in Figure 2e. Although not statistically significant, since the graph in Figure 2e is a Log2 scale, the mean difference between WT and F1 appears similar in magnitude to the differences seen in the co-culture experiment depicted in Figure 2f (which uses a more precise NGS based readout). Similarly for Figure 4, the data seems to be derived from n=1 experiment as reported in the figure legend. This is not enough to draw sound conclusions.

We apologize if this was not clear, but our experimental design does testing of include multiple edited clones in multiple biological replicates. For example, Figure 2 depicts multiple WT clones (e.g. A, B, WT1, WT1), V617F heterozygotes (H1, H2), V617F homozygotes (F1, F2, F3), and F617V (cH1, cF1, cF2, cF3, cF4). Each of these were tested in multiple biological replicates. For example, we conducted the growth assay shown in Figure 5e in 5 independent biological replicates. The number of replicates per experiment is detailed in each Figure legend.

3. Figure 3e is described in the manuscript (lines 329-332) as being conceptually similar to the co-culture experiment with HUDEP-2 cells. As this experiment was conducted, this is not the case, since methylcellulose assays read out predominantly single cell derived colonies when seeded with standard cell densities. One possible way to address this could be to sort single V617F cells into 96 well plates containing methylcellulose, and then sort single V617V cells into the same wells to ensure they are being cultured together and interacting via contact dependent and independent mechanisms.

The proposed co-culture experiment is quite interesting, and we spent quite some time trying to figure out how to set up something along these lines. The problem is identifying HSPCs with a defined edit for sorting. Figure 3e shows a bulk HSPC experiment, not clonal HUDEP-2s. HSPCs cannot be *in vitro* single cell cloned over long periods of time, and so we would need to somehow bulk edit these cells then find a way to sort them based on a genotyped SNP rather than a cell surface phenotype. We looked into repurposing technologies such as Millipore SmartFlare to accomplish this, but did not make progress. So far as we are aware, single cell separating HSPCs based on their genotype (e.g. V617F or V617V) is not possible with existing technologies. In fact, this problem the motivation behind recent advances in bulk single cell multi-omics approaches such as Genotyping of Transcriptomes (Nam et al 2020), which identify (but cannot separate) single cells based on their genotype. Hence, we are unfortunately not able to pursue the experiment as suggested by the referee.

4. The majority of patients with MPN are heterozygous for V617F, but most of the studies reported here are conducted with homozygous mutants. What as the phenotypic effects of V617F heterozygous HUDEP-2 clones? Do they still exhibit a competitive advantage in vitro? It would be interesting to conduct experiments as in Figure 3f with a 50/50 mixture of V617F and V617V ssODNs, in order to quantify differences in colony formation between V617V/V617F heterozygotes and V617V/V617V or V617F/V617F.

We apologize if this was not clear, but the phenotypes of HUDEP-2 V617F heterozygotes are shown in Figure 2c, Figure 2d, and figure 2g. The V617V results control for confounders from the process of editing rather than “memory” of DNA repair at an allelic locus, and so V617F/WT should be identical to V617F/V617V.

5. The manuscript would be greatly strengthened by in vivo xenograft data demonstrating the competitive advantage of JAK2 V617F CD34+ HSPCs compared to wild type.

This is a wonderful suggestion, and we began experiments along these lines in 2020. Unfortunately, the COVID-19 pandemic put a halt to these experiments and we have been unable to pursue them. We feel that the *ex vivo* data presented in the manuscript support a competitive advantage for JAK2 V617F cells, and have added Discussion points indicating that further *in vivo* xenograft data will be needed to follow up these conclusions (line 426-430).

Other points:

6. It would be interesting to know summary statistics for the HUDEP-2 targeting and genotyping. What percentage of V617F heterozygous clones harbored an indel in the other allele? Figure S2a uses different nomenclature (clone 1-7) than S2b and the majority of the paper (e.g. clone F1, H1, etc...), making it difficult to know which clones were depicted in the gel image.

Thank you for your suggestion. We agree that it could have been interesting to know the summary statistics for the V617F targeted HUDEP-2s. However at the time of the screening, we relied on RFLP assay to identify clones rather than sequencing. Clones that did not look promising by RFLP were discarded, and so we cannot go back and sequence them. However, we would presume that at least some of the heterozygous clones would harbor an indel on the other allele, as has been reported in several other papers and as suggested by the HSPC colony forming assay results in Figure 3f/g.

7. Due to the limited sensitivity of TIDE-based assays using deconvolution of Sanger traces, it would be helpful to use NGS amplicon sequencing to quantify indel frequencies of F617 sgRNA on WT alleles (Figure 2b).

Previous studies have shown that TIDE/ICE can be used to quickly characterize and quantify the indels generated during CRISPR-mediated gene editing in cell pools, and that the results derived from TIDE/ICE are almost as sensitive as NGS (Brinkman, et al PMID:25300484; Sentmanat, et al PMID:29343825). However, we understand your concerns regarding limited sensitivity of Sanger trances and TIDE/ICE-based assays, and we have has used NGS in most of our experiments (Figures 1c, 1d, 1e, 2f, 2g, 2h, 3b, 3c, 3e, 3f, S3c, S3d, S3e).

8. Findings from Figure S3a-c are similar to what has been reported in genome editing of HSPC literature, e.g. Genovese, P., Schiroli, G., Escobar, G. et al. Targeted genome editing in human repopulating hematopoietic stem cells. Nature 510, 235-240 (2014) doi:10.1038/nature13420 (suggest citing this study and others that find higher editing efficiencies in progenitors than phenotypic HSCs.

Thank you for your suggestion. We have included additional citations that report higher editing efficiencies in progenitor stem cells than HSCs (shown in line 269 in the manuscript).

9. HUDEP-2 flow cytometry (e.g. Figure S2g). Authors should use standard live/dead discriminating dye (e.g. DAPI or PI; or amine reactive fixable live/dead stains) rather than reporter expression (Kusabira-Orange), as dead/dying cells can display autofluorescence.

Kusabira-Orange with an absorption maximum at 548nm and emission peak of 561nm can be detected in the PE channel. We observed that dead/dying cells was clear of any autofluorescence in the PE channel and we also gated the cells on forward/side scatter to try to exclude any dead cells and debris. We have described this more in detail in our manuscript (lines 226-229).

Reviewer #1 (Minor Comments):

1. The term "electroporation" is preferable and more descriptive than the term "nucleofection" marketed by one particular manufacturer of equipment/reagents.

Thank you for pointing this out. We have changed the terms to electroporation throughout our manuscript.

2. The sentence beginning in line 112 is not very clear: "sgRNA-6 targets an intronic site...which could be less desirable for HDR." Is there data that shows the targeting efficiency of introns is lower than exons, or is there another issue the authors are suggesting?

We apologize that this was not clear. We solely meant that the intronic site is further away from the V617 coding site. When using ssODNs to stimulate efficient HDR, we and others have previously found that several factors including distance between sgRNA recognition site and the mutation, and the orientation, polarity, and length of donor ssDNA are important (line 114). Correspondingly in this manuscript, we have generated HDR by using sgRNA-4 or sgRNA-6 and have found that sgRNA-4 is preferable for generating the mutations of our interest (Figure 1c and 1d). sgRNA-4 is located right on top of the V617 site.

3. (line 121) "seed region" (or seed sequence) to our knowledge generally refers to miRNA. Suggest rewording or changing to "protospacer" instead.

Generally, it is standard in CRISPR field to refer to PAM-proximal 10-12 bases of the protospacer as seed region. Protospacer certainly includes the seed region, but does not refer exclusively to the seed region. (Cong, Ran, et al. 2013. *Science*; Jiang et al. 2013. *Nature Biotechnology*)

4. Figure S2g: Y-axis for flow plots not visible.

We have changed the plots to make the axes more visible.

Reviewer #2 (General assessment and major comments (Required)):

The MPNs are caused by driver mutations in JAK2, MPL and CALR. Numerous animal models have been created which recapitulate the disease. In addition, iPS cell lines have been derived from patients with the MPNs (eg Saliba et al PLoS 2013; Baumerister et al, Leukemia 2019). Finally, patient samples have been extensively studied to characterize the changes in hematopoietic cell development that are associated with these driver mutations.

In this manuscript Corn and colleagues report the use of CRISPR/Cas9 reagents to generate JAK2V617F mutant hematopoietic cells (HUDEP-2 and CD34+ cells) in vitro. They demonstrate that the presence of JAK2V617F leads to a competitive growth advantage, enhanced erythroid cell differentiation even in the absence of cytokines. They also show that they can correct the mutation by gene editing and revert the phenotypes. Their overall conclusion is that endogenously acquired JAK2V617F modifications may yield more subtle phenotypes as compared to transgenic overexpression models.

Major points

1. The issue with this paper is limited novelty and advancement to the field. There have been reports of many different models for the MPNs, including the well studied JAK2V617F knock-in model, iPS models generated by gene editing or derivation from patients, and of course patient samples themselves. The main conclusions of this manuscript essentially recapitulate what is already known.

We apologize if the novelty of our current work was not apparent. Our main focus was investigating the sufficiency of the JAK2 V617F mutation in a minimal system where the mutation is introduced to the endogenous locus with as few confounders as possible. Previous reports used many valuable, different models for investigating JAK2 V617F in MPNs. These included retroviral transduction, *in vitro* studies of primary PV patient samples and transgenic mouse models of MPNs. The mouse models have been extremely informative in confirming the role of mutated JAK2 protein in MPN pathology. However, the phenotypes were varied where some models developed mild or severe PV that progressed to myelofibrosis while other models simply developed ET (Lacout et al PMID 16670266; Wernig et al PMID 16478879; Zaleskas et al PMID 17183644; Tiedt et al PMID 18160670; Etheridge et al PMID 24469804). Some of these models may also be subject to problems inherent to retroviral-mediated transduction while transgenic model systems exhibit non-physiologic expression of the oncogene due to increased copy number and the use of exogenous promoters, and differences in signaling triggered by the murine EpoR when compared to human EPOR (Mullally et al PMID 20541703). Our data provide phenotypic data on precise “surgical” mutation of *solely* the V617 site in the endogenous locus.

2. The novel part of this paper lies in the correction of the JAK2V617F mutation in hematopoietic cells. The results certainly support the potential for gene editing in autologous transplants, but as the authors discuss, this approach will be challenging due to the need for complete (or near complete) correction. For this important part of the manuscript to be more compelling, the authors should obtain primary human MPN samples and demonstrate the feasibility of gene correction and show the degree of reversion of the in vitro phenotypes.

This is a great suggestion, and we initiated these studies in early 2020. However, the COVID-19 pandemic has stymied progress in this regard. Through a collaborator, we obtained CD34+ HSPCs from a patient who is a JAK2V617F heterozygote. Our initial attempts at editing revealed that these cells are far less tolerant to editing than HSPCs from healthy donors. We were in the process of optimizing editing in a patient context when the pandemic mostly shut everything down. Improving post-editing viability is a technicial barrier against which we have not been able to make significant progress due to continued problems caused by the pandemic. However, we see value in reporting our substantial results on introducing the V617F mutation and our early experiments on V617V/correction in model cells. We hope that the referee can agree, especially since the COVID-19 situation seems unlikely to lift in the next year.

Reviewer #2 (Minor Comments):

Minor points:

1. The color difference between the bars in figure 2b is difficult to see.

We apologize for making the colors difficult to distinguish. We have changed the colors to make it easier to see.

2. Figure 4a and 4b show data from an n=1 for all samples except GlyA. Why is the study limited to a single specimen? At least 3 should be provided regardless of the low population (%).

This is related to the COVID-19 shutdown.

Reviewer #3 (General assessment and major comments (Required)):

In an original report to eLIFE, Corn et al. report the use of CRISPR-Cas9 gene editing to introduce and repair the JAK2V617F MPN disease-initiating mutation in HUDEP and primary human CD34+ cells. The performed in vitro erythroid differentiated assays to link genotype to phenotype. The novelty of the work is that to my knowledge this is the first report of repairing the JAK2V617F mutation in primary CD34+ cells and as such provides proof of concept that this can be achieved.

The main criticism is that authors do not appear to have a detailed knowledge of MPN and there are a number of factual inaccuracies in the manuscript. All of the data was generated in vitro and in general the amount of data is relatively sparse (i.e. 3.5 figures). In my mind, the major strength of the paper is the ability to repair the JAK2V617F mutation, however this does not seem to be emphasized by the authors.

We are not certain exactly what the referee means when they say “factual inaccuracies”. None of the major or minor comments point these out, and so we are at a loss as to how to respond to this critique. We do note that the referee appears to have misconstrued the intention of our manuscript, and believed that we were attempting to answer an entirely different question (see response to first Major Comment below). If the referee thought we were addressing something different, then of course our manuscript seemed to fall short. We hope that clarifying the fundamental intent of our study and its experiments addresses many of the comments by the referee.

Major comments:

All the experiments are performed as if EPO is the only relevant cytokine. However, the EPOR is not expressed on HSC and in particular is not expressed on LT-HSC. Thrombopoietin, the ligand for MPL is the relevant ligand/receptor for assaying JAK2-dependent signaling in LT-HSC. G-CSF and GM-CSF are relevant for MPP and myeloid progenitor cell populations. If the authors wish to assay the impact of the JAK2V617F on HSC, EPO signaling is not the relevant signaling axis.

We apologize for the confusion. Our experiments were *not* designed to read out HSCs or progenitors. We use genome editing to introduce JAK2 V617F mutation into HSPCs, since these cells have been implicated as harboring the somatically acquired mutation in MPNs. We then ask how acquisition of this mutation at the endogenous locus affects differentiation to *erythroid* cells, which are most relevant for PV. Erythroid differentiation is normally EPO dependent, and a classic diagnostic for MPN and PV in particular is EPO-independent growth in methylcellulose. Hence our many experiments measuring erythroid markers in the presence and absence of EPO, with and without editing of the JAK2 mutation. We have attempted to make the intent of our manuscript more clear in the revised version. (line 235, 392-394, 437)

In Figure 2, there is inconsistency in the degree of pSTAT5 activation in JAK2V617F heterozygotes (i.e. comparable to homozygote in 2c, much less than homozygote in 2e). The authors state on page 7 that their data suggest that "the V617F allele is haplosufficient at the endogenous locus in hyperactivating JAK2 signaling" however it has been demonstrated in primary human MPN samples that EPO-independent growth (i.e. endogenous erythroid colony, EEC formation) is only seen in the context of homozygous JAK2V617F mutation (i.e. in polycythemia vera) and not in heterozygous JAK2V617F mutation (i.e. in essential thrombocythemia). See reference: PMID 17389763. Also, a growth advantage for JAK2V617F+ cells is only expected when the concentration of EPO is limiting (e.g. < 0.05 IU/ml). Based on the methods, the EPO concentration in these experiments was 3IU/ml and therefore a growth advantage for JAK2V617F cells would not be expected. See reference: PMID 17389763

We believe that the reviewer is referring to Figure 2d, rather than 2e (since 2e shows growth rather than Western blotting for p-STAT5). However, the referee seems to have mis-read these figures. We apologize if they were not clear. Figure 2c shows similar increases in pSTAT5 for the V617F heterozygotes and homozygotes (e.g. compare no-EPO light and dark red bars). The same is also true in Figure 2d, where the V617F heterozygotes and homozygotes have similar pSTAT5 (compare light red and dark red bars). Single allele reversion of a V617F homozygote into a heterozygote (V617F/V617V) also shows a similar level of pSTAT5. The greatly reduced pSTAT5 is solely in the double allele reversion of the V617F homozygote into a WT homozygote (V617V/V617V). In general, we see strong growth and molecular phenotypes in the context of single allele V617F mutation.

We apologize if EPO conditions were not clear, but the growth advantage experiments were not conduced solely in high concentrations of EPO. As stated in the Methods and in the legend to Figure 2, we compared competitive growth both with and *without* EPO.

Reviewer #3 (Minor Comments):

In the introduction, the authors discuss the order of acquisition of JAK2V617F and TET2 mutations and the consequential impact on MPN phenotype, however they do not reference the most relevant paper regarding this topic i.e. Ortmann & Kent et al. (PMID: 25671252)

Thank you for pointing this out. We have included the citation in the manuscript.

In the discussion, the authors state "The link between the prevalence of the JAK2 V617F allele and initiation of MPNs has thus recently become less clear, and it is possible that JAK2 mutation is associated with clonal hematopoiesis of indeterminate potential (CHIP) rather than causative of disease". This statement is factually incorrect. There is an abundant evidence that JAK2V617F mutation causes MPN, including the original four papers from 2005 describing the identification of the mutation as a recurrent driver mutation and more recently the NEJM paper by Grinfeld et al. indicating that in approx 40 - 50% MPN cases JAK2V617F is the sole mutation identified in MPN and in patients < 40 years old JAK2V617F always occurs the a sole MPN-causing mutation. See reference: PMID 30304655. The fact that JAK2V617F is a CHIP mutation provides further evidence that it is disease-initiating since alone is sufficient to engender clonal hematopoiesis. As with all CHIP mutations the prevalence of JAK2V617F CHIP is higher than the prevalence of JAK2V617F MPN, a fact which likely relates to factors that constrain and promote the expansion of JAK2V617F positive clones.

We appreciate the referee’s strong feelings about the role of JAK2V617F as a causative mutation. However, we feel that the existence of several primary research articles debating this point and asking whether it is instead associated with CHIP suggests that the link may not be as strong as initially supposed, and that this question is worthy of discussion. See Genovese et al NEJM 2014, Jaiswal et al NEJM 2014, Zink et al Blood 2017, Jaiswal et al NEJM 2017, Abelson Nature 2018, Hinds et al Blood 2016, Xie et al Nature Medicine 2014.
