# Supplementary material for: Genome editing to model and reverse a prevalent mutation associated with myeloproliferative neoplasms
Source: PLoS One. 2021 Mar 4;16(3):e0247858. doi: 10.1371/journal.pone.0247858 (PMC7932127; doi:10.1371/journal.pone.0247858)
Supplement: Attachment — Submitted filename: Response to reviewers.docx [file pone.0247858.s007.docx]

Reviewer #1: The authors reported on an array of experiments using Cas9-based reagents to create and reverse the JAK2 V617F mutation in an immortalized human erythroid progenitor cell line (HUDEP-2), CD34+ adult human hematopoietic stem and progenitor cells, and immunophenotypic long-term hematopoietic stem cells (LT-HSCs).

The first question was whether acquiring the mutation in human hematopoietic cell lineages is sufficient for clonal transformation. They found that : 1. the V617F allele is haplosufficient in hyperactivating JAK2 signalling; 2. the JAK2 V617F homozygous clones exhibited a very modest increase in growth rate over WT clones; 3. acquisition of the JAK2 V617F mutation leads to a competitive growth advantage over WT cells in a mixed ex vivo setting; 4. In the co-culture setting, JAK2V617F was over-represented in the presence of EPO, but the abundance of the JAK2 V617F allele was markedly enhanced in the absence of EPO; 5. There was no difference in differentiation between WT and either heterozygous or homozygous JAK2-V617F clones. They summarized these results by stating that JAK2V617F is sufficient to engender clonal expansion. But they could not claim on the basic question whether JAK2V67F is sufficient to initiate the phenotypically evident disease.

The role of JAK2V617F in clonal myeloproliferative diseases has been the aim of many different studies. Some of them arrived to the conclusion that on its own, JAK2V617F is insufficient to initiate disease (Hasan S, et al. Blood. 2013;122(8):1464-1477; Mullally A, et al. Blood. 2013;121(18):3692-3702; Li J, et al. Blood. 2014;123(20):3139-3151). Thus, the results of this study do not add a great bulk of novelty. However, the use of a minimal system in which the mutation is introduced to the endogenous locus with as few confounders as possible, makes the results worth of consideration.

A major comment is that a synthesis of the results, and which of them is confirmatory of previous evidence and which is new, is lacking, For example the results of phospho- STAT5 should be discussed in the context of very discordant results published in literature.

We thank Reviewer #1 for raising an important point about explaining our results in the context of current literature. As such we have updated our discussion section to more clearly explain what is new and what is confirmatory, as well as including the apt references they suggest. Specifically, please refer to lines 563-565 describing the insufficient role of JAK2 V617F in initiating full-blown MPN and 581-584 for descriptions of JAK2-STAT5 signaling.

A further part of the paper was on the effect of V617F mutation on in vitro differentiation. In the results section a long discussion is made about the protocol of editing. This part is difficult to understand by the non experts in the editing methods and we suggest to move it in the methods section.

We thank the reviewer for suggestions on how to clarify our manuscript. We have moved experimental details of the in vitro differentiation protocol and the list of surface markers used for immunophenotyping from the results section into the methods. Further, we have included a table to display some of the results that were previously described in the text (see Table S1d).

Reviewer #2: Baik and colleagues presented a genome-editing model to generate JAK2 p.V617F positive human cell lines as well as primary hematopoietic stem and progenitor cells.
They could show that p.V617F cells do not have an increased proliferation rate, but a highly competitive advantage in growth compared with the wild-type in a co-culture system. Furthermore, the Authors found that p.V617F allele is sufficient to increase STAT5 signaling even in the absence of EPO. The strength of these results is that they were not found in an artificial model system like retrovirally transduced murine HSCs. Several studies show a great variety of effects in transgenic mouse models and the development of myeloproliferative neoplasms or acute leukemia. Human genome-edited cells are good complement, or even an alternative to the animal models.
The manuscript show straight-forward hypothesis generating work, and complex result generating technical approaches were used (CRISPR, NGS, flow cytometry, western blotting). In my opinion the reviewer comments (Reviewer #1-3) were carefully and fully answered.
